# *In Vivo* Imaging Sheds Light on Immune Cell Migration and Function in Cancer

**DOI:** 10.3389/fimmu.2017.00309

**Published:** 2017-03-22

**Authors:** Tommaso Torcellan, Jessica Stolp, Tatyana Chtanova

**Affiliations:** ^1^Immunology Division, Garvan Institute of Medical Research, Sydney, NSW, Australia; ^2^Faculty of Medicine, St Vincent’s Clinical School, University of New South Wales, Sydney, NSW, Australia

**Keywords:** *in vivo* imaging, antitumor immune response, intravital microscopy, immune suppression, migration, cellular dynamics

## Abstract

There is ample evidence for both beneficial and harmful involvement of the immune system in tumor development and spread. Immune cell recruitment to tumors is essential not only for the success of anticancer immune therapies but also for tumor-induced immune suppression. Now that immune-based therapies are playing an increasingly important role in treatment of solid tumors such as metastatic melanomas, precise analysis of the *in vivo* contributions of different leukocyte subsets in tumor immunity has become an even greater priority. Recently, this goal has been markedly facilitated by the use of intravital microscopy, which has enabled us to visualize the dynamic interactions between cells of the immune system and tumor targets in the context of the tumor microenvironment. For example, intravital imaging techniques have shed new light on T cell infiltration of tumors, the mechanisms of cancer cell killing, and how myeloid cells contribute to tumor tolerance and spread. This mini-review summarizes the recent advances made to our understanding of the roles of innate and adaptive immune cells in cancer based on the use of these *in vivo* imaging approaches.

## Introduction

**Figure 1:**
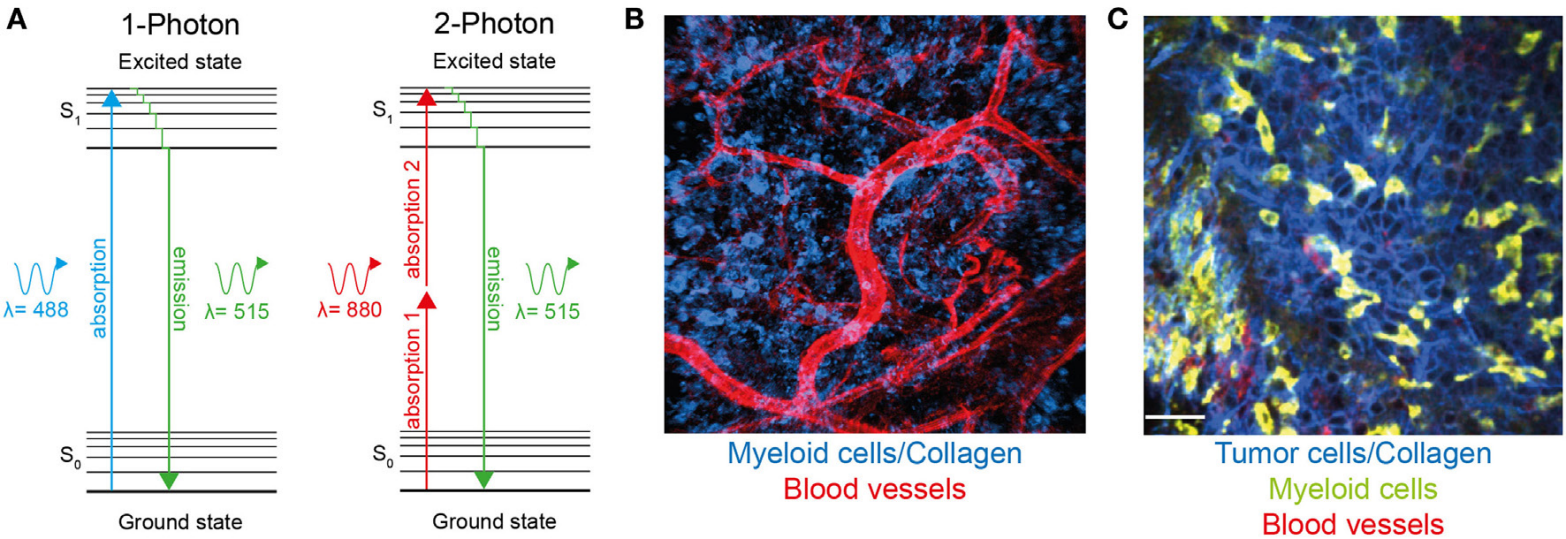
**Two-photon microscopy can be used to visualize immune cells and structures in normal tissue and within the tumor microenvironment**. **(A)** Jablonski diagram comparing one-photon and two-photon excitation. Excitation occurs as fluorophores are excited from the ground to the first electron state. While one-photon excitation occurs through the absorption of a single photon, near simultaneous absorption of two lower-energy photons *via* short-lived intermediate states is required for two-photon excitation. After either excitation, the fluorophore relaxes to the lowest energy level of the first state. The subsequent fluorescence emission processes for both relaxation modes are the same. **(B)** Two-photon microscopy used to visualize blood vessels (red) and Lysozyme M^+^ myeloid cells (blue) in murine skin. **(C)** A single optical section of a Lewis lung carcinoma (blue) infiltrated by lysozyme M^+^ myeloid cells (green), blood vessels (red) visualized using two-photon microscopy.

Immune infiltration is a common feature of most solid tumors, and the nature of these infiltrates has both prognostic and therapeutic implications. Now that immune therapies are gaining traction in the treatment of multiple types of cancer, it is becoming increasingly important to understand the complex interactions between immune cells and the tumor microenvironment. In particular, it is crucial to visualize these interactions *in situ* since the full complexity of the tumor microenvironment cannot be recapitulated in *in vitro* culture systems. Intravital imaging has proven to be an important tool to uncover the role of immune cells in tumor responses by revealing dynamics, interactions, spatial relationships, and distribution of leukocytes in tumor settings. Two-photon microscopy (Figure 1), as well as spinning disk and rapid resonant scanning confocal microscopy, provide low phototoxicity combined with high spatial resolution and allow for repeated scanning of tissue, thus providing temporal information about cellular migration and interactions.

Both lymphoid and myeloid compartments have been demonstrated to play important but frequently opposing roles in tumor responses. This mini-review summarizes some of the recent insights obtained with intravital imaging techniques into both positive and negative interactions between immune cells and tumor deposits *in vivo*, and how this approach is helping understand the role of non-malignant tumor components in tumor progression and responses to therapy.

## Visualizing Myeloid Cells in Cancer

Infiltration of tumors by cells from the myeloid lineage is usually associated with accelerated primary tumor growth and metastasis as well as poor prognosis (1). A recent study focusing on the timing of immune cell infiltration found that myeloid cell recruitment to the lung in response to metastatic B16 melanoma occurred in coordinated “waves,” beginning with the arrival of neutrophils within minutes of tumor cell appearance. Conventional monocytes, and finally macrophages, patrolling monocytes, and dendritic cells (DCs) followed neutrophil infiltration (2). A study in fluorescent translucent zebra fish larvae showed that neutrophils are able to recognize and interact with preneoplastic cells when they are still single cells or small clusters of clones (3). Macrophages were the second immune cell to be recruited to the preneoplastic clones, and the presence of macrophages was independent of neutrophil infiltration (4). While our understanding of the properties and functions of myeloid cells is complicated by the use of varying markers for identifying the cell populations involved, as well as plasticity associated with these cells types, the use of intravital imaging techniques has led to a clearer understanding of the different roles of tumor-infiltrating myeloid lineage cells in tumorigenesis.

### Neutrophils

It is becoming increasingly apparent that neutrophils, in addition to being the first immune cell subset to be recruited to sites of inflammation, also play an important role in cancer pathogenesis. While some antitumor functions for neutrophils have been reported (5, 6), recent evidence points to a role for them in promoting tumor spread and metastasis (7–10). Using intravital imaging, neutrophils have been shown to enhance adhesion of metastasizing tumor cells within the liver sinusoids (11). A subsequent study by Ferri and colleagues demonstrated that neutrophil extracellular traps (NETs), one of the mechanisms utilized by neutrophils to capture and destroy pathogens, can also play a role in sequestration of circulating tumor cells thereby promoting metastasis (12). In an *in vivo* mouse model, NETs induced by infection trapped intravenously injected circulating lung carcinoma cells leading to the formation of hepatic micrometastases. Furthermore, DNAse treatment or neutrophil elastase inhibition to disrupt NET formation prevented metastases, highlighting the role of NETs in tumor spread (12).

### Macrophages

Tumor-associated macrophages have been implicated in many different facets of tumorigenesis, largely as immunosuppressive cells associated with poor patient prognosis [reviewed in Ref. (13)]. One interesting imaging study has revealed that tumor-infiltrating macrophages in a mouse breast cancer model are the main immune subset targeted by bisphosphonate drugs. This finding may provide a mechanism whereby bisphosphonates improve disease outcome (14). Time-lapse recordings of macrophages labeled with fluorescent nanoparticles demonstrate that they cluster within tumors, show relatively low motility, and engage in prolonged interactions with tumor cells *via* cytoplasmic protrusions (15). Intravital microscopy has also provided important insights into some of the mechanisms used by macrophages to promote tumor growth and spread. For example, multiphoton imaging in mice has revealed that the interaction between macrophages and tumor cells establishes a microenvironment essential for the intravasation of tumor cells (16). Utilizing confocal intravital microscopy in combination with anti-colony-stimulating factor-1 receptor antibody treatment, it was possible to demonstrate both the depletion of tumor-associated macrophages and DCs, as well as the inhibition of their survival and the accumulation of cells within the tumor stroma (17). Furthermore, when treatment with the antibody was prolonged, it delayed growth of the primary tumor, decreased lung metastasis, and reduced tumor vascularity.

Notably, macrophages have been found at specific sites within tumors called “Tumor MicroEnvironment of Metastasis” (TMEM), which have been implicated in tumor cell intravasation (18). Using intravital high-resolution two-photon microscopy in a mouse mammary tumor model, Harney and colleagues showed that perivascular macrophages within TMEM were associated with increased vascular permeability and tumor intravasation (19). These findings applied not just to the mouse mammary cancer model, but to human xenograft models and human metastatic breast cancer as well, highlighting the enhancing role of macrophages in tumor metastasis.

Macrophages have also been associated with the development of metastatic lesions at distal sites. For instance, CCR2-expressing monocytes and macrophages can interact with tumor-derived CCL2 and aid the migration of macrophages to lung metastases in a breast cancer model (20). Furthermore, three-dimensional reconstructed confocal images of dissected mouse lungs (common site of metastasis in this model) showed significantly fewer tumor cells and macrophages after treatment with anti-CCL2 antibody. Significantly, a similar interaction was important for the development of lung metastases in human breast cancer patients, with high CCL2 expression and macrophage infiltration indicative of a poor prognosis (20). Likewise, in a recent study utilizing a mouse model of colorectal cancer, tumor-associated macrophages derived from CCR2^+^ monocytes played a crucial role in development of the extracellular matrix, revealing a novel pro-tumor mechanism for macrophages (21). Fluorescence lifetime imaging microscopy has been used to visualize macrophages within the stroma of mouse mammary tumors (22). In this study, the authors utilized the endogenous fluorescence of flavin adenine dinucleotide (FAD) and glycolytic-like nicotinamide adenine dinucleotide (NAD) to detect tumor-associated macrophages through imaging windows implanted in mouse skin. Macrophages infiltrating mammary tumors were identified by their unique metabolic signature of high FAD and NAD and thus could be distinguished from the surrounding tumor cells. Notably, since this imaging approach relies on endogenous fluorescence to visualize immune cells in tumors rather than transgenic or fluorescent labels, it has potential for clinical applications (22, 23).

### Dendritic Cells

Dendritic cells constitute an important link between the innate and adaptive immune systems. They take up and transport antigen from peripheral tissues to the lymph nodes or spleen where they present it to T cells. As the central regulators of adaptive immune responses, most subsets of DCs play a crucial role in T cell-mediated antitumor immunity [reviewed in Ref. (24)]. For example, two-photon imaging of OVA-expressing MCA101 tumors revealed that DCs are organized into a mesh-like pattern within the tumor, where they engage in antigen-dependent stable contacts with OVA-specific CD8^+^ T cells. Interestingly, T cell infiltration was restricted to DC rich areas, where, instead of promoting T cell immunity, the DCs may trap T cells and limit the antitumor function of these cells (25). Live imaging has also demonstrated a role for CCR7-dependent trafficking of intratumoral CD103^+^ DC from the tumor to draining lymph nodes (26). This migratory DC subset could either stimulate lymph node CD8^+^ T cells directly or transfer antigen to resident myeloid cells. Furthermore, CCR7 expression levels in human tumors directly correlated with CD141^+^ DCs and T cell infiltration, as well as improved survival in metastatic melanoma patients. This result links DC migration from tumors to draining lymph nodes with T cell infiltration and antitumor immunity.

Intravital imaging of DCs has revealed that in addition to their role in stimulating T cell responses, they also influence immune responses through interactions with other leukocytes subsets involved in tumor immunity. For instance, two-photon microscopy has showed that within draining lymph nodes, tumor-derived antigen is readily transferred from subcapsular macrophages to follicular DCs for presentation to B cells, indicating a potential role for DCs in inducing humoral responses, which are common in melanoma patients (27). Furthermore, *in vivo* imaging of leukemic B cells in a transgenic mouse model of B cell chronic lymphocytic leukemia showed that intratumor B cells can interact directly with follicular DCs, accelerating B cell proliferation and clinical progression (28).

Dendritic cells can also interact with natural killer (NK) cells, leading to their increased activation. Two-photon microscopy demonstrated that after lipopolysaccharide treatment, the extent of NK cell activation correlated with prolonged interaction with DCs in the lymph node. Once activated, these NK cells infiltrated a proximal tumor and impaired tumor growth (29). Thus, intravital microscopy has revealed several previously unappreciated aspects of the interplay between the tumor microenvironment and cells of the innate immune system.

## Cellular Dynamics of Lymphoid Cells in Tumor Responses

T cells constitute one of the key elements of the tumor microenvironment but due to the large number of distinct T cell subsets, their role in tumor immunity is especially complex. Some tumor-infiltrating T cell subsets, such as CD8^+^ cytotoxic T lymphocytes (CTLs), have important antitumor functions and their presence often correlate with a better prognosis (30). In contrast, suppressive T cell subsets, such as regulatory T (Treg) cells, impair antitumor responses and promote tumor growth, their presence within tumors therefore generally being associated with poor disease outcome (31, 32). Furthermore, T cell-based immune therapies aimed at either boosting the function of CTLs to combat cancer or inhibiting Treg cells that subdue antitumor responses represent some of the most promising recent developments in the treatment of solid tumors (33–35). Just as for myeloid cells, intravital imaging has provided important insights into the function of lymphoid cells in cancer that could not have been obtained using other methods.

### Cytotoxic T Lymphocytes

Given the prominent role that CTLs play in tumor destruction, it is unsurprising that their dynamics in antitumor responses have been the subject of intense investigation. Intravital microscopy has provided important clues about migration and function of these cells in tumor settings. For instance, *in vivo* imaging revealed that during the tumor regression stage, CTLs migrate in a random walk in the presence of their cognate antigen and engage in sustained interactions with intratumoral macrophages and tumor cells (36). Furthermore, antigen expression by tumor cells determined the depth of tumor infiltration by CTLs and their motility within tumors in a murine model of EL4 thymoma (37). Interestingly, during the early phase of tumor rejection, tumor-specific CTL migration was slow and frequently interrupted by interactions with antigen-expressing tumor cells. However, this migration became more rapid in the later phase of tumor rejection in areas of extensive tumor cell death. Intratumoral CTL motility was reduced in comparison to tumor-infiltrating NK cells, due to the long-lasting contacts between CTLs and their targets (38). The dynamics of tumor elimination by CTLs have also been visualized and quantitated using *in vivo* imaging (39). Adoptively transferred tumor-specific CTLs were shown to engage directly with individual tumor cells and then to kill them. Surprisingly, this process was estimated to take an average of 6 h, suggesting such a lapse in time might be one of the factors limiting the efficacy of antitumor immunity at least in this model. While factors that control intratumoral CTL migration are still being elucidated, the cell adhesion molecule CD44 has emerged as a critical regulator of this process. CD44 engagement stabilized T cell polarity and promoted T cell migration within tumors, which has the potential to lead to more efficient screening of tumor cells and immune surveillance (40).

To obtain longitudinal information about immune responses in developing tumors, Shrieber and colleagues implanted windows into the back skin of mice through which tumors could be repeatedly imaged. This enabled them to visualize T cell infiltration of tumors following the adoptive transfer of tumor-specific T cells. Their analysis revealed stromal interaction and vessel destruction (41). The tumor vessel destruction was initiated by perivascular T cells, while T cell interactions with the cross-presenting tumor stroma led to enhanced T cell effector function and bystander elimination of tumor cells.

Despite the fact that intravital microscopy is not currently feasible in patients, real-time imaging in unfixed sections of fresh human tumors has provided some insights into the dynamics of human tumor-infiltrating T cells *ex vivo* (42). CTL migration in the tumor stroma, where these T cells were more abundant, was slower compared to less populated tumor deposits. Interestingly, the collagen fibers in the tumor extracellular matrix affected intratumoral T cell distribution and migration, acting as both a physical barrier and a guidance structure for CTLs. This suggests that the extracellular matrix may influence tumor growth and could become the target of novel immunotherapies aimed at promoting T cell migration into tumors.

### Treg Cells

Regulatory T cells play a critical role in the suppression of antitumor immunity, and their dynamics and mechanism of action in cancer settings are of great interest. Analysis of Treg function in tumor-draining lymph nodes demonstrated that tumor-specific Treg cells inhibit CTL lytic functions without impairing CTL motility or their ability to form conjugates with target cells. Interestingly, inhibition of CTL function could be reversed by Treg removal (43).

Furthermore, Treg interactions with DCs in tumor-draining lymph nodes have been associated with DC death (44). Two-photon intravital microscopy revealed that this cell death was mediated by perforin and dependent on the presence of both the tumor and/or tumor antigens, suggesting this mechanism to be a pro-tumor response designed to limit the priming of CD8^+^ T cells. Consistent with this, Treg cell inactivation or depletion led to enhanced numbers of DCs and better T cell priming in draining lymph nodes.

The pro-tumor role of tumor-experienced Treg cell interactions with DCs was also investigated by Bauer and colleagues using two-photon microscopy (45). In this study, Treg cells that had encountered the tumor antigen in draining lymph nodes subsequently interacted with DCs within the tumor leading to the downregulation of costimulatory molecules CD80/86 on DC surface. Importantly, activation of tumor-infiltrating CTLs by Treg-conditioned DCs promoted CTL dysfunction *via* upregulation of coinhibitory receptors PD-1 and Tim-3. Thus, imaging studies have been instrumental in uncovering the mechanisms of Treg suppression of antitumor immune responses.

### B Cells

The role of B cells in cancer is still unclear. However, the antigen-presenting capability of activated B cells appears resistant to tumor microenvironment-induced immunosuppressive factors, such as IL-10 and TGF-β (46), suggesting that this immune subset may present an attractive target for immunotherapy. In an EL4 murine lymphoma model, adoptively transferred activated B cells migrated to tumor-draining lymph nodes where they interacted with T cells and induced antigen-specific CTL responses (47). Taken together, these findings highlight the potential of B cells as a cellular adjuvant for cancer immunotherapy.

## Concluding Remarks

Intravital microscopy is providing unique opportunities to observe immune cell migration and function *in situ* within both primary tumor deposits and secondary lymphoid organs where antitumor responses are initiated. Thus, we now have a better understanding of how CTLs find and kill their targets inside tumor deposits and how Treg cells can inhibit these processes, as well as the mechanisms utilized by myeloid cells to enhance cancer cell migration and spread. As immune therapies are poised to become a major weapon in cancer therapy, intravital imaging will be invaluable for monitoring *in vivo* effects of such therapies.

Another promising area for future investigations is visualizing the earliest interactions between immune cells and preneoplastic cells since this may provide insights into the early mechanisms that tumor cells utilize to alter immune cell functions. A better understanding of this process may lead to early intervention therapeutics or even prevention strategies.

**Figure 2:**
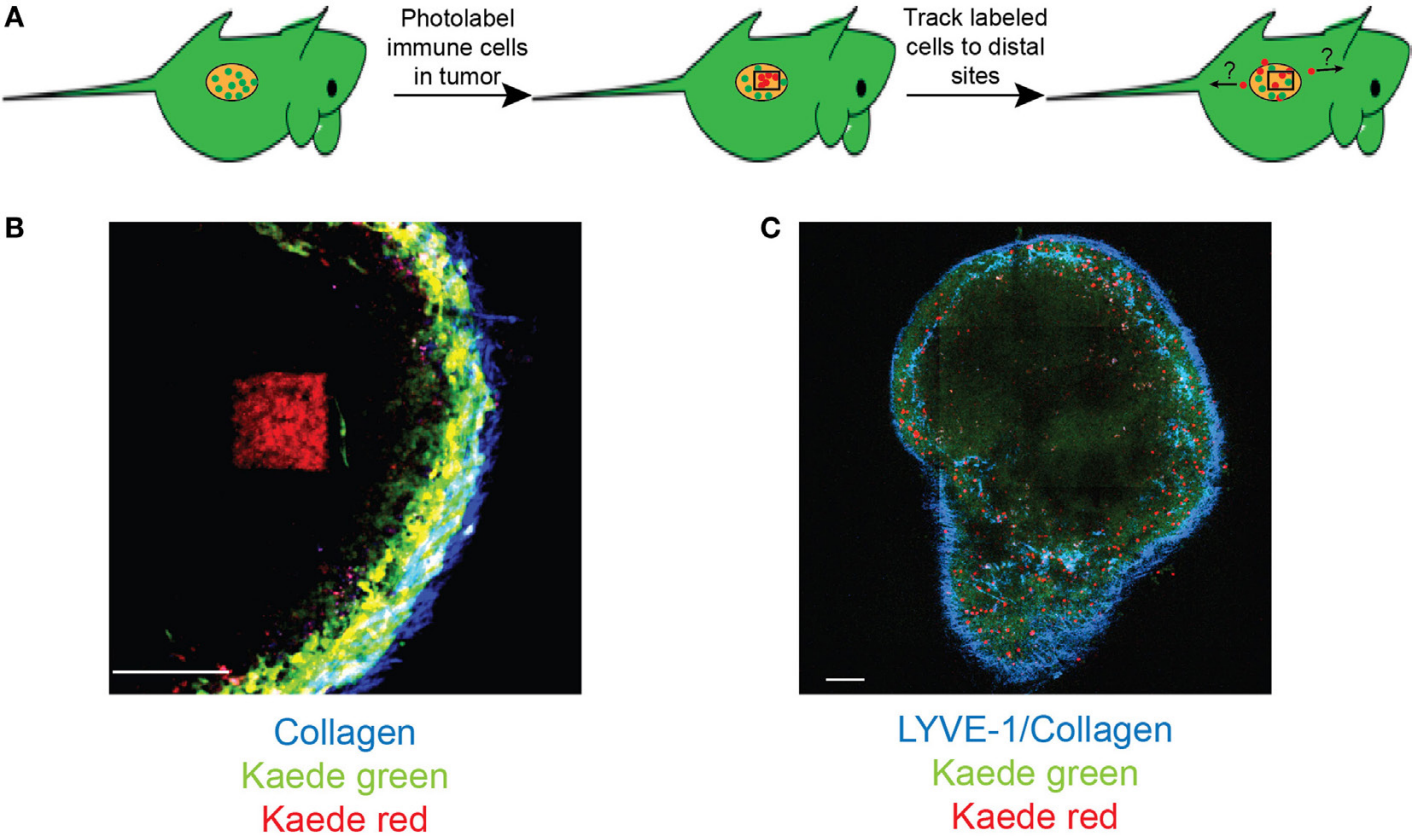
**Photolabeling extends the utility of intravital microscopy for analysis of immune cell migration in cancer**. **(A)** A schematic for labeling tumor-infiltrating cells. Two-photon microscopy is used to photoconvert cells within a specific region of interest within the tumor deposit. Microscopy and/or flow cytometry can then be used to detect photolabeled cells in distal organs. **(B)** An example of two-photon photoconversion of a single region (red) within a lymph node of a Kaede photoconvertible transgenic mouse. Non-photoconverted cells (green), LYVE-1^+^ lymphatics/collagen (blue). Scale bar represents 100 µm. **(C)** A single optical section of a murine lymph node containing photoconverted cells (red), non-photoconverted cells (green), LYVE-1^+^ lymphatics/collagen (blue). Scale bar represents 500 µm.

Although intravital imaging provides a dynamic view of the tumor-immune system interplay, analysis is still limited to a relatively short imaging window (typically 30–120 min) and a single organ or tumor deposit at any one time. The development of new tools, including photoswitchable reporter mice, which allow labeling of cells following exposure to light of specific wavelengths, has the potential to overcome these limitations. For instance, photolabeling using transgenic mice expressing Kaede fluorescent protein, which can be irreversibly converted from green to red upon exposure to UV or violet light, has already been successfully used by us (48) and others to monitor immune cell migration over large distances (49). When used in combination with two-photon microscopy, photoswitchable reporters allow for precise microanatomical labeling of selected regions of interest (50) and even individual cells (51, 52). This combination can be utilized to label cells within primary tumor deposits and track their fate, migration, and function both within as well as outside of the primary tumor (Figure 2). This approach will undoubtedly provide a powerful tool in understanding how immune cells migrate between lymphoid organs, primary tumors, and sites of metastasis.

The development of new fluorescent proteins and biosensors when combined with continuing improvements in imaging and analysis techniques will also shed further light on the complex dynamic interactions that occur *in vivo* between the immune system and solid tumors.

## Author Contributions

All authors listed have made substantial, direct, and intellectual contribution to the work and approved it for publication.

## Conflict of Interest Statement

The authors declare that the research was conducted in the absence of any commercial or financial relationships that could be construed as a potential conflict of interest.
